# A two-fold challenge: the experience of women of color in genomics

**DOI:** 10.1186/s13059-016-1075-0

**Published:** 2016-10-11

**Authors:** Ling-Ling Chen, Katrina G. Claw, Sohini Ramachandran

**Affiliations:** 1 Shanghai Institute for Biochemistry and Cell Biology, Chinese Academy of Sciences, 320 Yueyang Road, Shanghai, 200031 People’s Republic of China; 2 Department of Pharmaceuticals, University of Washington, 1959 NE Pacific Street, T163A, Seattle, WA 98195 USA; 3 Center for Computational Molecular Biology, Brown University, Providence, RI 02912 USA

## Abstract

What can be done to encourage and support women of color in STEM fields? *Genome Biology* spoke with three women of color who have had success in the area of genomics research.

## Introduction

The number of women of color in STEM fields lags behind those of white women. A recent report [1] found that 100 % of women of color experience bias both because of their gender and their race/ethnicity. *Genome Biology* spoke to Ling-Ling Chen, Katrina Claw, and Sohini Ramachandran about their personal experiences.

### Why did you decide to pursue a career in science?

**Sohini Ramachandran:** I was lucky to be exposed to academia as a career from a young age—my mother and father were both professors of mathematics and statistics. Growing up, I sat in their classrooms during my school vacations, drew on chalkboards during their faculty meetings, and puzzled over math problems their colleagues would pose to me. I also had great science teachers in junior high and high school, and worked in Professor Marcus Feldman’s laboratory during high school on a project that ultimately won fourth place in the 1998 Westinghouse (now Regeneron) Science Talent Search. These early experiences taught me that an academic career meant spending a lifetime learning, creating new knowledge, and working collaboratively; these are the reasons why I ultimately decided to become a scientist.

**Katrina Claw:** I don’t think I ever intentionally pursued a career in science. I began as an engineering major and quickly realized that it wasn’t for me. By chance, I took a human anatomy course in my second year of college, and it changed my whole trajectory. My career in academic research began when I started working in a research lab, and it was my initial fascination with genetic variation and interconnections that has taken me to where I am today.

I always knew that I was a part of something bigger and more connected than just myself. Growing up in an isolated, rural place like the Navajo Nation, you get to know your animals and environment pretty well. In the Navajo worldview, we see ourselves as a part of the land and everything as interconnected. An important Navajo concept is to *Hózhóogo naasháa doo* (‘Walking in Beauty’)—this refers to walking through your life in a balanced way—taking and giving in equal proportions.

**Ling-Ling Chen:** I gained basic biology and molecular biology training in China at Lanzhou University and the graduate school at the Chinese Academy of Sciences (CAS). I liked biology but wasn’t really considering a research career until I started my PhD at the University of Connecticut in 2004. My mentor was Gordon Carmichael, known for his studies on the polyomavirus life cycle and on RNA processing, editing, and function. When I joined the lab, Gordon let me explore new directions for the lab, perhaps because of my training in China. I had a feeling of ‘freedom’ in thinking and doing experiments my own way. The first several projects that I worked on didn’t work out, which made me somewhat hesitant about staying in academia at that time. So, I did an MBA in management during my ‘free’ time, thinking that the PhD–MBA dual degree would make me more competitive in the job market. The MBA required me to finish 19 courses and some case studies. In those two years, I worked from 7:30 a.m. to 6:30 p.m. in the lab every day, and then drove to business school, eating in the car; I often returned to the lab after class to finish experiments. As soon as I started business school, all my scientific projects seemed to work out! Eventually I thought this was because I didn’t have time for hesitation. These experiences were precious and kept me happy and fulfilled. During this process, I acquired a ‘can-do’ attitude and many impossible things just became manageable. Meanwhile, I also got exposure to another world, which allowed me to identify my enthusiasm for research and motivated me to stay in academia.

### Were there any perceptions, within and/or outside of your community, about your career prospects?

**SR:** My research advisors (Marc Feldman and John Wakeley), as well as my dissertation committee members and Fellows at the Harvard Society, were always very encouraging about my career prospects. I was also very ambitious. I articulated pretty early on that I wanted to become a professor, even though I knew very little about what that entailed. I talked a lot about job searches with people whose careers I wanted to emulate, especially recent faculty hires in my field, and that helped me set goals to work towards, including traveling to conferences and proposing symposia, and actively publicizing my publications. I’ve always visualized both my short- and long-term goals, and that has been a very positive influence on my career trajectory.

**KC:** I didn’t fully understand ‘research’ or ‘academia’ until I had years of lab experience and conferences behind me. So it’s always with a little perplexity that people view my career prospects because a scientific research career is not something that Native American communities are generally exposed to. Yet, my family and community understand the tremendous amount of dedication and hard work that went into obtaining a bachelor, a doctoral degree, and being away from our homelands. I have so much support and encouragement relating to my education from my family, community, and the larger Native American community. I’m so thankful for that, and even if they don’t understand what I’m doing, I still strive to represent my family and community in a way that values my upbringing and traditional knowledge.

**LC:** Perhaps I had learnt that women should have their own careers from my grandma; she didn’t have the opportunity to receive an education, like most other women of her generation in China, but she always wanted to have her own career. She tried very hard to let her five children (four of whom, including my mom, are female) receive the best education possible, and all five went to college, which was not common in China in the 1980s. Her attitude completely changed the family’s social status and greatly influenced me to acquire knowledge and pursue an independent career.

### What were your experiences early on in your career?

**SR:** Research is a solitary road in many ways, and I think that building a large cast of mentors, senior faculty, and peers was very important during my training and faculty career. I found that so many people in academia have great advice to offer about various challenges, including research and lab management, teaching, and grant writing. My peers from my graduate program still play a huge role in my life—we invite each other to give seminars and over time we have developed active collaborations.

**KC:** I went to Arizona State University for my dual bachelor degrees in Biology and Anthropology. Although ASU has a strong Native American community, and I had some family in the area, moving to a large city was a huge culture shock and I was homesick for much of my first year. I was at the top of my class in high school, but I was wholly unprepared for the rigorous calculus and physics courses that I signed up for in my first year. I edged by in my courses with ‘Cs’, which eventually led to me losing all of my scholarships by the end of my sophomore year. My family could not help with tuition costs and I took out many loans, trying to scrape by. At that point, my parents were trying to support four children in college and technical schools. In my junior year, I was fortunate enough to get accepted into the Minority Access to Research Careers (MARC) program, which changed my life. I chose a lab to work in (and had amazing mentors), and the MARC program assisted me with tuition and a monthly stipend and planted the seed of graduate school in my mind. After I graduated from ASU, I attended the Post-baccalaureate Research Education Program (PREP) at ASU. PREP enabled me to take GRE-prep and graduate-level courses to show that I could excel, and the research I did in my lab led to a first-authorship publication. By the end of the second year of the PREP program, I knew I wanted to pursue a graduate degree.

I was accepted into all seven graduate schools that I applied to and chose to attend the University of Washington, in Seattle. I didn’t know anyone and missed my family, friends, community, and the southwest so much. I was at a top-tier research institution with peers from Harvard and Stanford, and everyone had so many big ideas and confidence. I was at my lowest point—I felt like an imposter and that I didn’t deserve to be there. Eventually, I formed a small community that I could rely upon, including a supportive advisor and departmental faculty, who encouraged me in my work with people of color at the University.

Despite this, I did feel isolated—people did not always understand where I came from and sometimes were not sensitive to this. A lot of the issues that we studied were far removed from the majority of my peers, such as the potential for using genomics to alleviate health disparities. ‘Health disparities’ is not a word I throw around; it is deeply personal and important to me—many in my community have type 2 diabetes and many others have died of cancer, alcoholism, or depression. It’s the same all over the country with other Indigenous groups. It is not only related to genetics—it has to do with a lack of access to resources such as food, water, education, and jobs and the historical trauma inflicted upon Indigenous, and many other, communities. Because of this, I pursued many initiatives throughout my graduate school years so that students like me would not have to feel the same way.

**LC:** I spent four and half years in graduate school at UConn. My experiences there were full of joys and hard work. As a foreigner, I first spent some time adjusting to living in Connecticut, both socially and academically, but the training in molecular biology in CAS prior to UConn had greatly facilitated the academic transition. In Gordon’s lab, after the failure of several projects, I began to study the function of Alu elements, which constitute more than 10 % of all of the DNA in the human genome. I found that inverted repeated Alu elements in the 3′ untranslated regions (UTRs) of mRNAs act to retain mRNAs in particular nuclear substructures called ‘paraspeckles’. Then I found that the key to Alu-element-mediated gene regulation is a long noncoding RNA (lncRNA) called NEAT1 that itself serves as a scaffold to organize the paraspeckles, which are nuclear storage sites for mRNAs that are in the cytoplasm. These findings represented a novel paradigm in gene regulation.

Before my PhD oral defense, I had applied for independent funding, which supported 100 % of my salary and science for two years following my PhD. I stayed in the same lab for postdoctoral training but worked largely on my own to pursue new directions. I was promoted to Assistant Professor in residence at the UConn Health Center one year later and then joined the Shanghai Institute of Biochemistry and Cell Biology, CAS, in early 2011.

### What have been your biggest challenges and greatest opportunities in your career?

**SR:** The most influential events in my career were all accidents, in that I couldn’t have predicted what big opportunities would come my way. The biggest challenge in my early faculty career was recruiting my first lab members. It took a few years to find people with the right background for my research program who also wanted to work with a junior faculty member; however, through multiple fantastic accidents, I ended up building my lab, and it is a privilege to work with each of my lab’s members. Another wonderful accident was meeting my husband, the historian Jeremy Mumford, while he and I were both postdocs at the Michigan and Harvard Society of Fellows, respectively. We didn’t live in the same state until we got married. Maintaining two academic careers has involved sacrifice, and my research and teaching benefit a lot from Jeremy’s perspectives as a historian. We are really pleased to now both have tenure-track jobs at Brown University, with wonderful advocates in our chairs, deans, and colleagues. It took a lot of work to achieve this goal and we seriously entertained moving multiple times in our careers in order to achieve it.

**KC:** By far, the biggest challenge has been overcoming culture shock and acclimatizing to new settings where I’m often the only Native American and/or person of color in the room/department/program/school. It can be isolating, lonely, and stressful. Imagine going through graduate school, or living in a city, without ever feeling completely comfortable in your environment. Before college, I lived and grew up on the Navajo Nation in the southwestern US, where more than 95 % of my peers and community are Navajo and only a small percentage are non-Native. In my community, we all shared a lot of the same understandings, values, and culture. Going to university and living in a city were culture shocks for me. I often felt like I couldn’t relate with the typical student experience and that I was an outsider; I was also under constant stress to maintain a balance between my career, family, culture, and traditions. I was the third person in my large extended family to get a bachelor’s degree and I’m the first person in my family and community to get a PhD. I was very lucky to find communities of color in graduate school, through my participation in the University of Washington Society for the Advancement of Chicanos and Native Americans in Science (SACNAS) Student Chapter and my work with the Seattle Native Clear Sky Youth Council and the larger Native American community in Seattle. These connections provided me with a community that supported me throughout my graduate school career and beyond.

The greatest opportunities in my academic career have been the opportunity to get an education and to pursue various research questions. There are so many promising young Native American students who are never exposed to these opportunities or who don’t have the support to finish the academic programs that they start. Not everyone has the knowledge or support system in place to navigate the financial and bureaucratic aspects of a higher education and it is especially important to provide this kind of support for first-generation college students. I have been very privileged in many ways: I have parents, and an extended family, who expected me to get a higher education, and they have supported me in many ways. I had older siblings who went to college before me and paved the way. Higher education has given me so many opportunities—I was able to travel the world for 8 months on the UW Bonderman travel fellowship, and, in 2011, I attended the 61st Annual Meeting of Nobel Laureates in Lindau, Germany, where I met many world-renowned Nobel prize winners. As I continue to progress in my field, it is simply amazing to realize that I have the opportunity to create my own research program, in which I plan to work in partnership with American Indian/Alaska Native/Indigenous peoples on projects that matter to those communities, which I hope will lead to increased research capacity for many communities. My future research in genomics and health disparities is only one part of a bigger picture in which I envision there being more Native Americans and Indigenous people in higher education and positions of leadership all over the country.

**LC:** I think that the decision to study for the MBA as a second degree was a challenge; however, it didn’t hurt my scientific career but instead helped me a lot in the way of thinking and doing multiple tasks at the same time. I think one great opportunity in my career was the independent funding I received from the State of Connecticut Stem Cell Research Fund right after I received my PhD in 2009, which allowed me to begin exploring new and bold directions. In 2009, perhaps one of the most exciting discoveries in molecular biology was the widespread expression of lncRNAs. I worked on the lncRNA NEAT1 and the unique properties of its 3′ end motivated me to search for additional novel types of lncRNAs by developing methods to visualize and characterize non-polyadenylated RNAs. This work has led to the discovery of several classes of RNA species in my lab.

### In your opinion, what can be done to encourage more women of color to move into STEM fields?

**SR:** I think women of color need more role models in STEM—not just faculty who are women of color but also more-diverse trainees and faculty members who are invested in training people to go on to successful careers in STEM. Another missing piece is getting to know faculty members personally. My undergraduate education at Stanford University emphasized close contact with faculty, through meals in the dining hall, seminars with capped enrolments, and social events that focused on career paths. I had many professors in college who were first-generation college students, but I would never have known that without hearing their life-stories. I wish more universities built infrastructure to help students learn how and why professors become professors.

**KC:** I think that we need to expose girls to science at a young age, have more-visible role models, and also have culturally inclusive and applicable science. People of color have been doing science in their communities for millennia, and it’s time that we bring this into the picture (i.e., Indigenous knowledge). When we teach science, why don’t we talk about how indigenous people used their own knowledge of genetics and relationships to cultivate and grow corn (and so many other plants) for thousands of years? There are so many examples of Indigenous science. This is especially important in Native American communities, where research has been misused and role models are hard to find. Throughout my academic career, it has been hard for me to find a mentor or role model with a similar background who I could aspire to…there are a handful of Native American faculty in the STEM fields right now. I definitely see a trend where more Native American professionals are moving into STEM, but there needs to be a concerted effort to provide support for this community. Yes, departments can accept a ‘diverse’ cohort of students initially, but, if the support and community are not there for people of color, it just makes a difficult program that much more difficult to succeed in. Advisers, faculty, and peers need to be cognizant of the adversity, micro/macro-aggressions and the lack of support that students of color face daily in their institutions.

Many students of color enter programs with the mind-set of eventually returning to their home communities, and the current academic pipeline is seemingly discouraging of this ideal. I’ve been cautioned by many people about limiting myself to a certain geographic region when I’m ready to apply for faculty positions, especially in light of current funding and job competitiveness. While I understand this, for me this is not something that I can easily compromise on because my whole family and culture is in the southwest. Things are changing, and we all make our own paths. I’m hopeful that, with additional training and creativity, I can still do the work that I’m passionate about.

**LC:** I don’t see a particular hurdle for women of color in the STEM fields in China, from a societal perspective in big cities. In my institute, the female lab-heads increased from 12 in 2010 to 19 in 2016 among 77 independent labs. Additionally, the numbers of newly entered female graduate students has been equal to or higher than men in the past several years. However, the disparity in these figures indicates that many female scientists still leave active academic research early in their careers.

In general, women have more social duties than men and therefore need more support and self-confidence. For myself, my mentors have meant a lot to me, were supportive, and made my early career extremely enjoyable. Gender was never an issue since it was really about the science for them. Their mentoring and support allowed me to gain self-confidence and self-belief throughout my training. I also found that senior female role models in a field tend to attract more women. For instance, there are great female scientists in the field of RNA biology—Joan Steitz is a pioneer in the study of small, noncoding RNA molecules; Lynne Maquat is a pioneer in the study of RNA quality control and also spent her career supporting young women in the sciences; Narry Kim and Mikiko Siomi have made tremendous contributions to our understanding of microRNAs and Piwi-interacting RNAs (piRNAs), respectively. These successful women greatly influenced my perception of my career prospects in graduate school and in my early career. Because of women like them in RNA research, younger women like me don’t face a dearth of role models and encouragement as scientists. Finally, family support is also important to encourage women to move into and stay in STEM fields.

## Abbreviations

CAS: Chinese Academy of Sciences
lncRNA: Long noncoding RNA
MARC: Minority Access to Research Careers
piRNA: Piwi-interacting RNA
PREP: Post-baccalaureate Research Education Program
SACNAS: Society for the Advancement of Chicanos and Native Americans in Science
STEM: Science, technology, engineering and mathematics
UTR: untranslated region
